# Prevalence and Characterization of *Escherichia coli* and *Salmonella* Strains Isolated from Stray Dog and Coyote Feces in a Major Leafy Greens Production Region at the United States-Mexico Border

**DOI:** 10.1371/journal.pone.0113433

**Published:** 2014-11-20

**Authors:** Michele T. Jay-Russell, Alexis F. Hake, Yingjia Bengson, Anyarat Thiptara, Tran Nguyen

**Affiliations:** Western Center for Food Safety, University of California Davis, Davis, California, United States of America; Beijing Institute of Microbiology and Epidemiology, China

## Abstract

In 2010, Romaine lettuce grown in southern Arizona was implicated in a multi-state outbreak of *Escherichia coli* O145:H28 infections. This was the first known Shiga toxin-producing *E. coli* (STEC) outbreak traced to the southwest desert leafy green vegetable production region along the United States-Mexico border. Limited information exists on sources of STEC and other enteric zoonotic pathogens in domestic and wild animals in this region. According to local vegetable growers, unleashed or stray domestic dogs and free-roaming coyotes are a significant problem due to intrusions into their crop fields. During the 2010–2011 leafy greens growing season, we conducted a prevalence survey of STEC and *Salmonella* presence in stray dog and coyote feces. Fresh fecal samples from impounded dogs and coyotes from lands near produce fields were collected and cultured using extended enrichment and serogroup-specific immunomagnetic separation (IMS) followed by serotyping, pulsed-field gel electrophoresis (PFGE), and antimicrobial susceptibility testing. A total of 461 fecal samples were analyzed including 358 domestic dog and 103 coyote fecals. STEC was not detected, but atypical enteropathogenic *E. coli* (aEPEC) strains comprising 14 different serotypes were isolated from 13 (3.6%) dog and 5 (4.9%) coyote samples. *Salmonella* was cultured from 33 (9.2%) dog and 33 (32%) coyote samples comprising 29 serovars with 58% from dogs belonging to Senftenberg or Typhimurium. PFGE analysis revealed 17 aEPEC and 27 *Salmonella* distinct pulsotypes. Four (22.2%) of 18 aEPEC and 4 (6.1%) of 66 *Salmonella* isolates were resistant to two or more antibiotic classes. Our findings suggest that stray dogs and coyotes in the desert southwest may not be significant sources of STEC, but are potential reservoirs of other pathogenic *E. coli* and *Salmonella*. These results underscore the importance of good agriculture practices relating to mitigation of microbial risks from animal fecal deposits in the produce production area.

## Introduction

Foodborne disease illnesses caused by pathogen contamination of fresh produce are being recognized in greater numbers in the United States (U.S.) and abroad [1], [2]. An analysis of Centers for Disease Control and Prevention (CDC) data on reported foodborne illnesses from 1973 to 1997 indicated that outbreaks associated with fresh produce accounted for 6% of all reported foodborne disease outbreaks in the 1990s compared with just 0.7% in the 1970s [3], [4]. A more recent survey of CDC outbreak data from 1998–2008 showed these numbers are still rising, with 46% of foodborne illnesses being attributed to produce and 22% specifically attributed to leafy greens [5]. While norovirus infections transmitted downstream during post-harvest handling are likely the major driver of these statistics, reports of fresh produce-associated outbreaks from zoonotic agents potentially spread by domestic and wild animal reservoirs in the pre-harvest environment are clearly contributing to this disease burden [6], [7].

**Figure 1:**
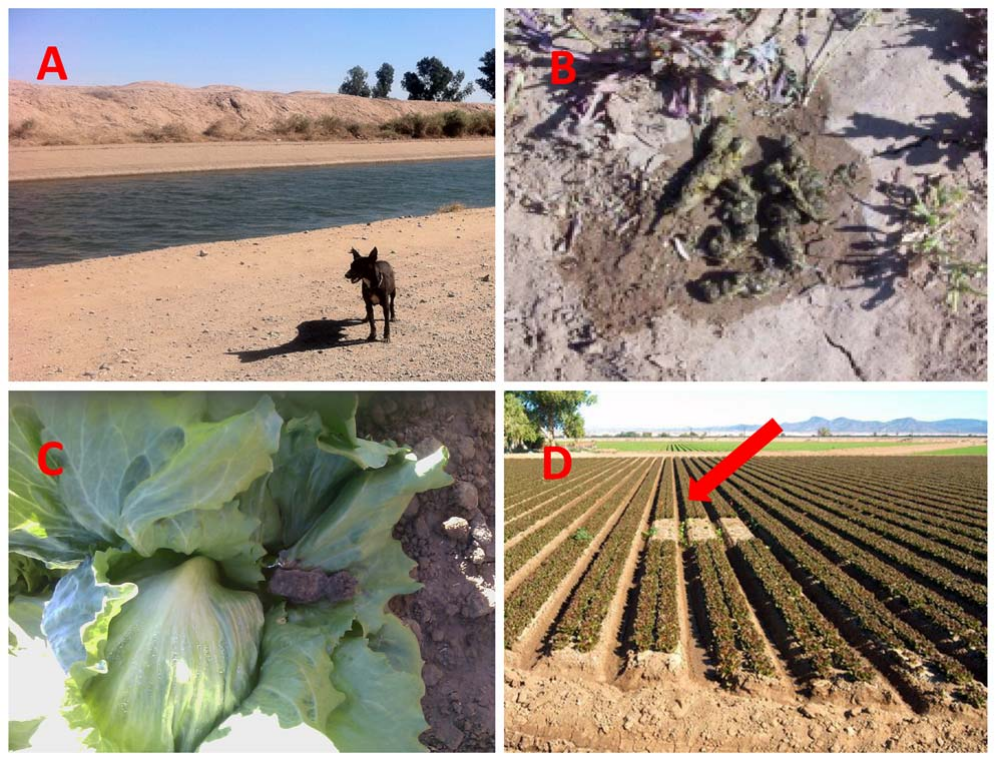
Examples of animal intrusions into produce production areas of the desert southwest: a stray dog traveling next to an irrigation canal in northern Mexico (A); coyote feces adjacent to a lettuce field in southern California (B); dog feces on a lettuce plant in southern Arizona (C); areas of intentionally destroyed lettuce crop (arrow) following evidence of animal intrusion.

Approximately 90% of commercial lettuce produced for the U.S. market is grown in two major produce production regions that rotate seasonally [8]: the Salinas Valley in the central California coast (April through October) and the desert southwest at the U.S.-Mexico border (November through March). The desert southwest growing region includes Yuma, Arizona, California's Imperial Valley, and northern Mexico. The role of domestic animals and wildlife as potential sources and transmitters of zoonotic bacterial pathogens to lettuce and other leafy greens and agriculture water has been studied at length in the central California coast [9], [10], [11], [12], [13]. There is limited information, however, on the importance of animal reservoirs in the pre-harvest bacterial contamination of fresh produce in other parts of the country [14]. The desert presents unique pre-harvest food safety challenges including urban encroachment where produce fields and irrigation canals may be adjacent to housing developments and recreation vehicle (RV) parks. In addition to concerns about human sources of foodborne pathogens near leafy green production areas of the desert, growers report problems with unleashed, free-roaming domestic dogs (*Canis familiaris*) entering their fields. Off-leash or stray dog intrusions into produce fields and the surrounding production area may result in damage to crops and destruction of potentially contaminated plants (Figure 1). Growers also report frequent coyote (*Canis latrans*) sightings and signs (tracks, scat, feces) on roads adjacent to produce fields where tractors and other equipment are used.

In spring 2010, an outbreak of *Escherichia coli* O145:H28 infections involving 27 confirmed and 4 probable case-patients from 5 states was linked to Romaine lettuce grown in southern Arizona [15], [16]. This was the first known leafy green-related Shiga toxin-producing *E. coli* (STEC) outbreak traced to the desert growing region. Based on investigations by the U.S. Food and Drug Administration (FDA), pre-harvest contamination of irrigation canals possibly due to sewage runoff from a nearby RV park could have caused the contamination, although no laboratory-confirmed environmental source of the outbreak was determined [17]. Following this outbreak, the present study was funded as a Center for Produce Safety “Rapid Response Project” with the purpose to determine prevalence and characterize pathogenic *Escherichia coli* and *Salmonella* strains isolated from dog and coyote fecal samples collected in the southwest desert during the 2010 to 2011 leafy green vegetable growing season.

## Methods

### Ethics Statement

Permission to access privately owned lands was obtained from the produce companies enrolled in the study. Animal shelter administrative directors in the U.S. and Mexico approved participation in the study. Dog fecal samples from the shelter in Mexico were transported by vehicle across the Mexico-US border by one of our industry collaborators. A permit for importation of dog feces was not required per the United States Department of Agriculture (USDA) Animal and Plant Health Inspection Service (APHIS) “Animal and Animal Product” import guidelines (#1102 Feline and Canine Material). Wildlife scientific collection permits and university animal care and use approval were not necessary in this study because fecal samples were collected from the ground and no animals were handled.

### Sampling

Three animal shelters were enrolled in the study, one each in Yuma, Arizona, Imperial Valley, California, and northern Mexico. These facilities were chosen because animal control officers had worked historically with leafy green growers in the region to remove stray dogs from agriculture fields. We aimed to sample once monthly during the desert southwest leafy greens growing season (November to March) with a goal to collect ∼300 samples based on sample size calculations. Due to limits in the number of impounded dogs available each month and logistics with the shelter personnel, each facility was sampled six times spread variably from November 3, 2010 to May 5, 2011 (Table 1).

**Table 1:** Monthly prevalence of atypical enteropathogenic *Escherichia coli* (aEPEC) and *Salmonella enterica* isolated from coyote and dog fecal samples, southwestern desert, November 3, 2010 through May 5, 2011.

			No. positive/No. tested (%)^a^						
Source	Animal	Location	Nov	Dec	Jan	Feb	Mar	May	Total
**aEPEC**									
	**COYOTES**								
		Arizona	0/10	0/11	1/11 (9.1)	0/7	0/0	0/0	1/39 (2.6)
		California	0/10	3/16 (18.8)	0/13	0/11	1/14 (7.1)	0/0	4/64 (6.3)
		Subtotal	0/20	3/27 (11.1)	1/24 (4.2)	0/18	1/14 (7.1)	0/0	5/103 (4.9)
	**DOGS**								
		Arizona	0/16	1/21 (4.8)	0/0	0/17	3/45 (6.7)	0/25	4/124 (3.2)
		California	1/24 (4.2)	3/18 (16.7)	0/23	1/18 (5.6)	1/17 (5.9)	0/0	6/100 (6.0)
		Mexico	0/30	0/28	1/27 (3.7)	2/49 (4.1)	0/0	0/0	3/134 (2.2)
		Subtotal	1/70 (1.4)	4/67 (6.0)	1/50 (2.0)	3/84 (3.6)	1/62 (1.6)	0/25	13/358 (3.6)
		**Total**	**1/90 (1.1)**	**7/94 (7.4)**	**2/74 (2.7)**	**3/102 (2.9)**	**2/76 (2.6)**	**0/25**	**18/461 (3.9)**
***Salmonella enteric***									
	**COYOTES**								
		Arizona	6/10 (60.0)	2/11 (18.2)	5/11 (45.5)	0/7	0/0	0/0	13/39 (33.3)
		California	5/10 (50.0)	7/16 (43.6)	3/13 (23.1)	3/11 (27.3)	2/14 (14.3)	0/0	20/64 (31.3)
		Subtotal	11/20 (55.0)	9/27 (33.3)	8/24 (33.3)	3/18 (16.7)	2/14 (14.3)	0/0	33/103 (32.0)
	**DOGS**								
		Arizona	0/16	2/21 (9.5)	0/0	0/17	2/45 (4.4)	0/25	4/124 (3.2)
		California	4/24 (16.7)	1/18 (5.6)	1/23 (4.3)	0/18	3/17 (17.6)	0/0	9/100 (9.0)
		Mexico	13/30 (43.3)	1/28 (3.6)	3/27 (11.1)	3/49 (6.1)	0/0	0/0	20/134 (14.9)
		Subtotal	17/70 (24.3)	4/67 (6.0)	4/50 (8.0)	3/84 (3.6)	5/62 (8.1)	0/25	33/358 (9.2)
		Total	28/90 (31.1)	13/94 (13.8)	12/74 (16.2)	6/102 (5.9)	7/76 (9.2)	0/25	66/461 (14.3)

a The Arizona shelter was sampled twice in March and in May. The California shelter was sampled twice in November. The shelter in Mexico was sampled twice in December and twice in February.

A standardized questionnaire was used to collect demographic data (location and date found, breed, sex, age, and reason the dog was impounded) from records at the facilities. Dog fecal samples (n = 358) were collected by industry cooperators after training by University of California, Davis (UC Davis) veterinarians in aseptic fecal sample collection, storage and shipping. Freshly deposited feces from animals caged individually at the U.S. shelters were taken from the kennel floor using a sterile tongue depressor and placed in a sterile 227 gram fecal cup with a snap-cap lid (National Scientific Supply Co., Claremont, CA). Dogs at the shelter in Mexico were caged in groups, thus there was potential for cross-contamination. To minimize the risk of cross-contamination, care was taken to collect only freshly deposited feces from individual dogs impounded in the past 24 hours.

Fresh coyote fecal material (n = 103) found on the roads in and up to 1 mile from leafy green vegetable fields located in Yuma, Arizona and Imperial Valley, California were collected by industry cooperators using a sterile tongue depressor and fecal cup as described above. Industry personnel routinely survey fields for animal intrusions and have training in the identification of sign (sightings, tracks, feces) of wildlife species including coyotes common in agriculture areas. In order to ensure the fecal material was fresh, the sites were walked by grower personnel the evening prior to sampling, and any existing coyote feces was removed. The fields were then surveyed again at dawn the following morning to collect fresh feces. Wildlife trail cameras (Cuddeback Digital, Green Bay, WI), physical sightings, and other sign were used to confirm the presence of coyotes at the study areas.

Samples were shipped to UC Davis overnight on blue-ice on the day of collection and processed on the day of arrival at the laboratory (approximately 24 hours).

### Laboratory

Our overall goal was to culture Shiga toxin-producing *E. coli* belonging to serogroups STEC O103, O145, O157, O26, and non-typhoidal *Salmonella enterica* using a combined pre-enrichment step followed by immunomagnetic separation (IMS), selective plating, latex agglutination, and PCR or biochemical confirmation as described below. Isolates were then characterized by presence of virulence factors, genetic relatedness, and antibiotic resistance.

#### Pre-enrichment

Initially, non-selective pre-enrichment for the simultaneous culture of STEC and *Salmonella* was performed by adding 10 grams of feces to 100 mL of universal pre-enrichment broth (UPB; Difco, Becton Dickinson, Sparks, MD) and incubating for 20 hours at 35°C using a protocol modified in our laboratory for dog fecal material [18]. One milliliter of enriched UPB was then transferred to 9 mL tryptic soy broth (TSB; Becton Dickinson, Sparks, MD) for STEC detection and incubated for 2 hours at 25°C with shaking at 100 rpm (Innova 44, Eppendorf North America, Hauppauge, NY), followed by 8 hours at 42°C with shaking at 100 rpm, then at 6°C without shaking until processing the following day [10], [19]. One milliliter of enriched UPB was also transferred to 9 mL of buffered peptone water (BPW; Hardy Diagnostics, Santa Maria, CA) for *Salmonella* detection and incubated for 24 hours at 37°C with shaking at 50 rpm [11]. In 2011, we discontinued the use of the UPB pre-enrichment step to streamline the protocol. Instead, pre-enrichment was performed by adding 10 grams of feces directly into a WhirlPak bag containing 100 mL TSB followed by the same incubation parameters as just described [10], [19]. Spiking experiments comparing the UPB and TSB pre-enrichment methods revealed no statistical difference (p = 0.32) in recovery of STEC or *Salmonella* (data not shown). As such, we completed this study using the streamlined protocol without the UPB step.

Aliquots of the primary enrichment broths were mixed with sterile glycerol to a final concentration of 14.3%, and stored at −20°C [11].

#### *Escherichia coli*

IMS using Dynal anti-*E. coli* O157, O26, O103, and O145 beads (Invitrogen, Grand Island, NY) was performed on TSB enrichment broths with the automated Dynal BeadRetriever (Invitrogen) per the manufacturer's instructions. Following incubation and washing, 50 µL of the resuspended beads were plated onto Rainbow agar (Biolog, Hayward, CA) with novobiocin (20 mg/L) and tellurite (0.8 mg/L) (MP Biomedicals, Solon, OH) and streaked for isolation [10], [19]. Another 50 µL were plated onto Sorbitol MacConkey Agar (BD Becton, Sparks, MD) with cefixime (0.05 mg/L) (USP, Rockville, MD) and tellurite (2.5 mg/L), streaked for isolation, and incubated at 37°C overnight. *E. coli* O157:H7 RM1484 and three non-O157 STEC strains (O103, O145, O26) were used as positive controls to observe the expected phenotype on the selective agars. Up to 10 colonies exhibiting characteristic morphology were subcultured to both agar types and incubated again at 37°C overnight for purification. Up to four pure colonies were then streaked onto Luria-Bertani (LB; Fisher Scientific, Waltham, MA) agar and incubated at 37°C for 24 hours. Suspect colonies from LB agar were screened using ImmuLex commercial latex slide agglutination assays (Statens Serum Institut, Denmark) with pooled STEC antisera (*E. coli* OK O antiserum to detect EPEC and STEC) followed by O-group specific antisera (O103, O145, O157, O26) according to the manufacturer's instructions. All assays included a negative control (saline) to check for non-specific agglutination. Two bacterial colonies from each positive plate were banked onto Cryobeads (ProLab Diagnostics, Round Rock, TX) and stored at −80°C until characterized further.

All isolates (n = 278) positive for STEC O-groups by latex agglutination were submitted to the Pennsylvania State University *E. coli* Reference Center to confirm O-type using a multiplex PCR that detects eight major STEC O-groups (O26, O45, O103, O111, O113, O121, O145, O157) as described previously [20]. H-antigens were identified by the same lab using PCR-RFLP [21]. The isolates were also tested by PCR at the reference laboratory for the presence of virulence factors including *stx*1, *stx*2, *eaeA*, and *hlyA* genes [22]. A subset of aEPEC isolates (n = 12) positive for the *eaeA* gene, but not belonging to the 8 STEC O-groups identified by the reference laboratory's multiplex PCR, were serotyped conventionally by agglutination reactions against antisera developed for each of the O serogroups [20].

Because all isolates were Shiga toxin-negative despite many belonging to STEC-associated O-groups, a retrospective analysis of banked TSB enrichment broths (n = 461) frozen at -20°C with glycerol was performed at the United States Department of Agriculture's Agricultural Research Service (ARS) Western Regional Research Center laboratory. Briefly, template DNA was prepared by boiling followed by detection of *stx*1 and *stx*2 virulence genes by multiplex qPCR; the method was validated previously for screening TSB pre-enrichment broths from environmental samples including coyote feces [19].

#### *Salmonella*

IMS using anti-*Salmonella* Dynabeads (Invitrogen, Grand Island, NY) was performed on BPW broths as described previously using the Dynal BeadRetriever (Invitrogen) [11]. Following incubation and washing, 100 µL of separated broth was further enriched in 3 mL Rappaport-Vassiliadis Soya Peptone (RVS; Difco, Becton Dickinson, Franklin Lakes, NJ) broth for 48 hours at 42°C [23]. Ten microliters of RVS broth were then plated on xylose lysine deoxycholate (XLD; Difco, Becton Dickinson, Franklin Lakes, NJ) agar and incubated at 37°C overnight. Samples with growth of hydrogen sulfide positive colonies were confirmed for *Salmonella* by performing biochemical profiles (triple sugar iron, urea, citrate, and lysine decarboxylase) on up to six individual colonies from each XLD agar. *Salmonella* Enteritidis ATCC BAA1045 was used as a positive control to observe the expected phenotype. The same colonies used for biochemical profiling were also streaked onto LB agar and incubated at 37°C overnight. Two bacterial colonies from each positive plate were banked onto Cryobeads (ProLab Diagnostics, Round Rock, TX) and stored at −80°C. Serotyping using the Kauffmann-White scheme was conducted by the United States Department of Agriculture (USDA) National Veterinary Services Laboratories in Ames, Iowa [24].

#### Pulse field Gel Electrophoresis

*E. coli* and *Salmonella* isolates were retrieved from frozen storage at −80°C and clonal relationships were assessed by pulsed-field gel electrophoresis (PFGE) according to the CDC's PulseNet standard procedure using *Salmonella* Braenderup ATCC BAA664 as the molecular size standard [25]. Briefly, bacterial isolates were suspended in buffer containing 10 mM Tris pH 8 and 10 mM EDTA for DNA isolation. DNA was digested in enzyme buffer with restriction enzyme *Xba*I. Images were analyzed, and the similarity among different strains was characterized using Bionumerics version 7.1 software (Applied Maths, Austin, TX). Pattern comparisons were made using the software cluster analysis tool and confirmed by visual examination to assign pulsotypes [26], [27].

#### Antimicrobial Susceptibility testing

Frozen *E. coli* and *Salmonella* isolates were thawed and streaked onto trypticase soy agar (TSA; Difco, Becton Dickinson, Franklin Lakes, NJ) with 5% sheep blood agar, then incubated at 37°C for 24 hours. The broth microdilution method for antimicrobial susceptibility testing was performed in accordance with the Clinical and Laboratory Standards Institute (CLSI) guidelines [28], [29]. Isolates were evaluated for susceptibility to 12 antimicrobial drugs (ampicillin, amoxicillin/clavulanic acid, ceftriaxonem azithromycin, chloramphenicol, sulfisoxazole, cefoxitin, kanamycin, streptomycin, trimethoprim/sulfamethoxazole, tetracycline, ceftiofur) using the National Antimicrobial Resistance Monitoring System (NARMS) Gram negative tray (Trek Diagnostic Systems, Westlake, OH). *E. coli* ATCC 25922, *E. coli* ATCC 35218, and *Pseudomonas aeruginosa* ATCC 27853 were used as quality control organisms for MIC determination in accordance with CLSI guidelines. Breakpoints guidelines were adopted from the NARMS report, with the exception of azithromycin, for which no breakpoints have been published [30]. Based on a recent publication proposing an epidemiologic cut-off for wild-type *Salmonella* of ≤16 µg/ml, isolates with MIC values >16 µg/ml were considered resistant to azithromycin for the purpose of this study [31].

### Statistical Analysis

WinEpi online software (http://www.winepi.net/uk/index.htm) was used to calculate sample size with a confidence level set at 95% and a population size of 1,000–10,000. Prevalence estimates were based on data from a longitudinal study of coyote populations in the central California coast in 2008–2010 [11], [19], with 1% for *E. coli* O157, 5% for non-O157 STEC, and 10% for *Salmonella enterica*; using these estimates, the required number of samples would be 258–294 for *E. coli* O157, 57–59 for non-O157 STEC, and 29 for *Salmonella* detection. Based on the expected low prevalence of *E. coli* O157, our goal was to collect at least 300 fecal samples.

Data were entered in Microsoft Excel 2007 spreadsheets and exported for analysis in STATA (Stata 11.0, College Station, TX). McNemar's chi-square test was used to compare the sensitivities of pre-enrichment methods (UPB and TSB) and O-group testing methods (latex agglutination, multiplex PCR). Univariate logistic regression was conducted to identify statistical associations between enteric pathogen status and covariates (age, sex, etc.). Covariates with p-values equal or less than 0.20 were considered for inclusion in an exact logistic regression model. A p-value ≤0.05 was used to detect significantly associated factors for pathogen presence in the regression model.

## Results

### Prevalence and risk factors

A total of 461 fecal samples were collected from November 3, 2010 through May 5, 2011, including 358 domestic dog and 103 coyote fecals (Table 1). Descriptive characteristics of the shelter dog population are shown in Table 2. Shiga toxin-producing *E. coli* was not detected in any fecal samples, but aEPEC (*eaeA*+) strains were isolated from 13 (3.6%) dog and 5 (4.9%) coyote fecal samples. *Salmonella* was detected in 33 (9.2%) dog and 33 (32.0%) coyote fecal samples.

**Table 2:** Summary of population characteristics from domestic dogs sampled in a southwest United States and northern Mexico produce production region, November 3, 2010 through May 5, 2011 (N = 358).

Demographic	Number Sampled (%)
**Shelter**	
Arizona	124 (34.6)
California	100 (27.9)
Mexico	134 (37.4)
**Reason Impounded**	
Stray	297 (83.0)
Other^a^	44 (12.3)
Unknown	17 (4.7)
**Age**	
Puppy	58 (16.2)
Adult	279 (77.9)
Unknown	21 (5.9)
**Sex**	
Male	165 (46.1)
Female	186 (52.0)
Unknown	7 (2.0)
**Breed**	
Chihuahua/Mix	41 (11.5)
Labrador/Shepherd Mix	56 (15.6)
Pit Bull Terrier/Mix	60 (16.8)
Other	37 (10.3)
Unknown	140 (39.1)

a Includes all dogs born in shelter, relinquished by owner, confiscated from owner, or dogs being kept for quarantine or treatment purposes.

Univariate analysis revealed no significant association between the relative number of aEPEC or *Salmonella* positive and negative dogs by age, gender, breed, or reason that the dog was impounded. There was a significant difference in the number of dog fecal samples positive for *Salmonella* by shelter location. Exact logistic regression revealed that, after adjusting for age, the odds of a dog from the shelter in Mexico being *Salmonella* positive was 4.88 (95% CI: 1.60, 20.31, P <0.01) times higher than for dogs at the Arizona shelter. In November 2010, a higher seasonal prevalence of *Salmonella* was observed at the California and Mexico shelters, primarily due to a specific serovar (Senftenberg) as described below.

For coyote fecal samples, univariate logistic regression showed no significant difference in the odds of aEPEC or *Salmonella* isolation in fecal samples collected in Arizona compared with California (OR = 1.10, 95% CI: 0.43, 2.78, P = 0.99). There was a significantly lower odds of *Salmonella* isolation from coyote scat sampled in February (OR = 0.16, 95% CI: 0.04, 0.70, P = 0.02) and March (OR = 0.1, 95% CI: 0.02, 0.80, P = 0.03) compared with November; but, unlike dogs, this seasonal observation was not due to any specific serovar.

### Phenotypic and molecular characterization of *E. coli* isolates

We screened initially for suspect STEC isolates by using O-specific (O103, O145, O157, O26) IMS and commercial latex agglutination. A total of 278 isolates presumptively belonging to these four serogroups were submitted to the Pennsylvania State University *E. coli* reference laboratory for confirmation using their multiplex PCR that detects 8 major STEC O-groups [20]. We found discordance between the IMS-latex agglutination classification of O-groups compared with the multiplex PCR (Table 3). Specifically, if the multiplex PCR at the reference laboratory is used as the standard, the sensitivities of O-specific latex agglutination (31.2% for O103, 8.5% for O145, and 26.3% for O26) were significantly different from the multiplex PCR method (p <0.01).

**Table 3:** Comparison of results from Shiga toxin-producing *E. coli* (STEC) O-group-specific (O103, O145, O157, O26) commercial latex agglutination screening tests and a multiplex PCR confirmatory test to detect 8 major STEC O-groups (O103, O111, O113, O121, O145, O157, O26, O45) used to serotype isolates cultured from fecal samples by selective enrichment and serogroup-specific (O103, O145, O157, O26) immunomagnetic separation (IMS).

		Multiplex STEC PCR								
Latex agglutination	No. isolates^a^	0103	0111	0113	0121	0145	0157	026	045	Other
O103	199	62	0	0	0	0	2	1	0	134^b^
O145	59	0	0	3	0	5	0	0	0	51^c^
O157	1	0	0	0	0	0	0	0	0	1
O26	19	0	0	0	0	0	0	5	0	14
Total	278	62	0	3	0	5	2	6	0	200^d^

a All isolates were *stx*1 and *stx*2 negative.

b Ten *eae*+ isolates classified as O103 by latex agglutination screening and negative by multiplex STEC PCR belonged to serotypes O114 (n = 1), O123 (n = 2), O126 (n = 1), O128 (n = 1), O167 (n = 1), O64 (n = 1) and O- (n = 3) using O-antisera.

c Two *eae*+ isolates classified as O145 by latex agglutination screening and negative by multiplex STEC PCR belonged to serotypes O153 (n = 1) and O- (n = 1) using O-antisera.

d Serotyping using O-antisera was not performed on isolates (n = 187) negative for virulence factors and not belonging to STEC O-groups identified by multiplex PCR.

All 278 isolates were negative by PCR for genes encoding *stx*1 and *stx*2. Additionally, 461 frozen fecal TSB-enrichment broths were negative using a multiplex qPCR assay to detect *stx*1 and *stx*2 genes [19]. Isolates (n = 187) lacking any virulence factors and not belonging to one of the eight serogroups identified by the reference laboratory's multiplex PCR were not further characterized. Among the remaining 91 isolates, a total of 29 different *E. coli* serotypes were identified (Table 4). Excluding clones from the same samples, there were 18 isolates comprising 14 serotypes with genes encoding *eaeA,* including one (O26:H11) with both *eaeA* and *hlyA* genes; these isolates were classified as aEPEC (Table 4) [32], [33]. There was more diversity among serotypes from coyotes compared with dogs. Specifically, 11 of 15 coyote fecal isolates were different serotypes with none being dominant. In contrast, almost half of the dog samples contained two dominant serotypes that were negative for virulence markers, O103:H16 and O103:H49 (Table 4).

**Table 4:** Serotypes and virulence factors of *Escherichia coli* strains isolated from dog and coyote fecal samples, southwestern desert, November 3, 2010 through May 5, 2011.

	Source			Virulence Factor		
Serotype^a^	Coyote	Dog	Total	*stx1*/*stx2*	*eaeA*	*hlyA*
**aEPEC**						
O-: H2	1	0	1	-	+	-
O-: H8	0	1	1	-	+	-
O-: H25	0	1	1	-	+	-
O114: H8^b^	0	1	1	-	+	-
O123: H+	0	2	2	-	+	-
O126: H9	0	1	1	-	+	-
O128: H2	1	0	1	-	+	-
O145: H34	0	3	3	-	+	-
O153: H21/36	0	1	1	-	+	-
O157: H+	2	0	2	-	+	-
O167: H9	0	1	1	-	+	-
O26: H11	0	1	1	-	+	+
O26: H8	1	0	1	-	+	-
O64: H19	0	1	1	-	+	-
**Subtotal**	5	13	18			
**OTHER**						
O103: H-	1	0	1	-	-	-
O103: H2	1	4	5	-	-	-
O103: H7	0	7	7	-	-	-
O103: H9	0	2	2	-	-	-
O103: H16^b^	0	11	11	-	-	-
O103: H19	1	0	1	-	-	-
O103: H21	1	0	1	-	-	-
O103: H21/36	2	1	3	-	-	-
O103: H40/44	2	1	3	-	-	-
O103: H43	0	1	1	-	-	-
O103: H49^b^	2	26	28	-	-	-
O113: H4^b^	0	3	3	-	-	-
O145: H11^b^	0	2	2	-	-	-
O26: H2	0	1	1	-	-	-
O26: H32^b^	0	3	3	-	-	-
**Subtotal**	10	62	72			
**TOTAL**	15	76	91			

a O-, O antigen non-determinant.

b Twelve dog fecal samples contained two different serotypes including O103:H16/O113:H4 (n = 6); O26:H32/O103:H49 (n = 5); and O114:H8/O145:H11 (n = 1).

**Figure 2:**
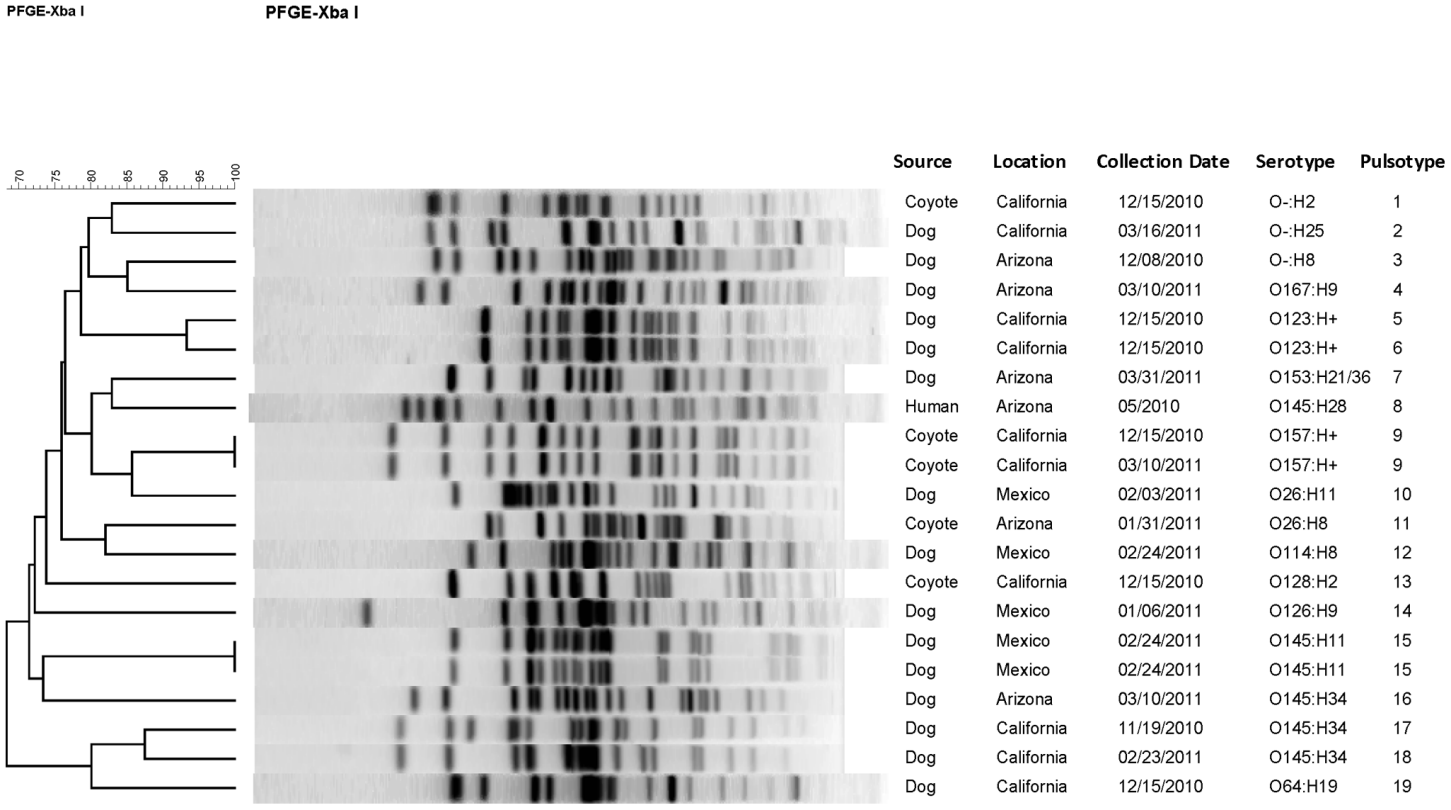
*Escherichia coli* (*XbaI* restriction) pulsotypes of 18 aEPEC isolates and 2 non-pathogenicirulent *E. coli* O145:H11 isolates from dog and coyote fecal samples in the southwest desert produce growing areas of Arizona, California, and northern Mexico, November 3, 2010 through May 5, 2011. A human clinical *E. coli* O145:H28 outbreak strain associated with a Romaine lettuce-related outbreak traced to Arizona in May 2010 is also shown.

A total of 17 pulsotypes (PT) were found among 18 aEPEC strains from dogs and coyote feces (Figure 2). Two non-pathogenic *E. coli* O145:H11 isolates were included in the dendogram for comparison with the *E. coli* O145:H28 human clinical strain (PT-8) associated with the 2010 outbreak linked to Romaine lettuce. Of note, *E. coli* O145 strains isolated from dog feces during the study had different H types (O145:H11 and O145:H34) and were genetically unrelated to the 2010 outbreak strain based on PFGE analysis (Table 4, Figure 2).

All aEPEC isolates were tested for antibiotic resistance, and 6 (33.3%) were found to be pansusceptible to the antimicrobial drugs we used in the NARMS panel (Table 5). One isolate from coyote and 3 isolates from dog feces were resistant to two or more antibiotics. Two aEPEC isolates, serotypes O167:H9 and O114:H8, from dog feces collected in Arizona and Mexico, respectively, were resistant to four antibiotics including ampicillin, ceftriaxone, chloramphenicol, tetracycline (O167:H9), and chloramphenicol, sulfisoxazole, streptomycin, and trimethoprim/sulfamethoxazole (O114:H8).

**Table 5:** Antimicrobial resistance patterns among 18 atypical enteropathogenic *Escherichia coli* (aEPEC) and 66 *Salmonella enterica* isolates from coyote and dog fecal samples, southwestern desert, November 3, 2010 through May 5, 2011.

Drug resistance pattern^a^	Source (No. of isolates)			Serotype or antigenic formula^b^
**aEPEC**	**Coyote (n = 5)**	**Dog (n = 13)**	**Total (% of all isolates)**	
Pansusceptible	2	4	6 (33.3)	O-:H2
				O-:H25
				O126:H9
				O145:H34
				O26:H8
FIS	2	6	8 (44.4)	O-:H8
				O123:H+
				O128:H2
				O145:H34
				O153:H21/36
				O157:H+
				O26:H11
				O64:H19
FIS-STR	1	0	1 (5.6)	O157:H+
FIS-TET	0	1	1 (5.6)	O123:H+
AMP-AXO-CHL-TET	0	1	1 (5.6)	O167:H9
CHL-FIS-STR-SXT	0	1	1 (5.6)	0114:H8
***Salmonella***	**Coyote (n = 33)**	**Dog (n = 33)**	**Total (% of all isolates)**	
Pansusceptible	29	29	58 (87.9)	Aqua
				Barranquilla
				Drac
				Duisburg Enteritidis
				Javiana
				Livingstone; Montevideo Muenchen
				Newport
				Oranienburg Sandiego
				Senftenberg Typhimurium Typhimurium var 5-
				II 47:b:1,5
				III 17:z29:-
				III 62:z36:- III_40:z4, z32:- III_48:g, z51:-III_48:i:z; IV
				44:z36:-
				IV 47:l,v:e,n,x
AMP	0	1	1 (1.5)	Enteritidis
STR	1	1	2 (3.0)	Typhimurium
				IV Rough O:autoagglutinate
XNL	1	0	1 (1.5)	Sandiego
AXO-TET	1	1	2 (3.0)	Mbandaka IV 44:z36:-
AMP-STR-SXT	0	1	1 (1.5)	Senftenberg
AMP-AUG2-AXO-CHL-SXT	1	0	1 (1.5)	Newport

a AMP, ampicillin; AUG2, amoxicillin/clavulanic acid; AXO, ceftriaxone; AZI, azithromycin; CHL, chloramphenicol; FIS, sulfisoxazole; FOX, cefoxitin; KAN, kanamycin; STR, streptomycin; SXT, trimethoprim/sulfamethoxazole; TET, tetracycline; XNL, ceftiofur.

b O-, O antigen non-determinant.

### Phenotypic and molecular characterization of *Salmonella* isolates

Overall, 29 different *Salmonella enterica* serovars were identified with 46 (70%) of 66 isolates belonging to subspecies Group I (Table 6). Two dominant serovars, Senftenberg and Typhimurium comprised 58% of the isolates from dog samples. In contrast, no predominant *Salmonella* serovars were identified among strains isolated from coyotes. A significant association was observed between serovar Senftenberg and the date of sample collection (P = 0.03), with 10 of the 14 *S.* Senftenberg isolations occurring in the month of November, primarily from the shelter in Mexico.

**Table 6:** Subspecies and serovars of *Salmonella* isolated from dog and coyote fecal samples, southwestern desert, November 3, 2010 through May 5, 2011.

		Source (No. isolates)		
Subspecies (Group)	Serovar or antigenic formula	Coyote	Dog	Total
*Enterica* (I)	Aqua	1	0	1
	Barranquilla	1	0	1
	Derby	0	1	1
	Drac	1	0	1
	Duisburg	1	0	1
	Ealing	0	1	1
	Enteritidis	0	2	2
	Javiana	2	0	2
	Livingstone	0	1	1
	Mbandaka	0	1	1
	Montevideo	1	0	1
	Muenchen	2	0	2
	Newport	3	0	3
	Oranienburg	0	1	1
	Sandiego	2	0	2
	Senftenberg	0	14	14
	Typhimurium	5	5	10
	Typhimurium var. 5-	0	1	1
*Salmae* (II)	47:b:1,5	1	0	1
*Arizonae* (IIIa)	17:z29:-	3	0	3
	35:z29:-	0	1	1
	62:z36:-	1	0	1
	40:z4, z32:-	3	0	3
	48:g, z51:-	0	3	3
*Diarizonae* (IIIb)	48:i:z	2	0	2
	50:r:z	1	0	1
*Houtenae* (IV)	44:z36:-	1	1	2
	47:l, v:e,n,x	2	0	2
Unknown	Rough O:autoagglutinate	0	1	1
Total		33	33	66

**Figure 3:**
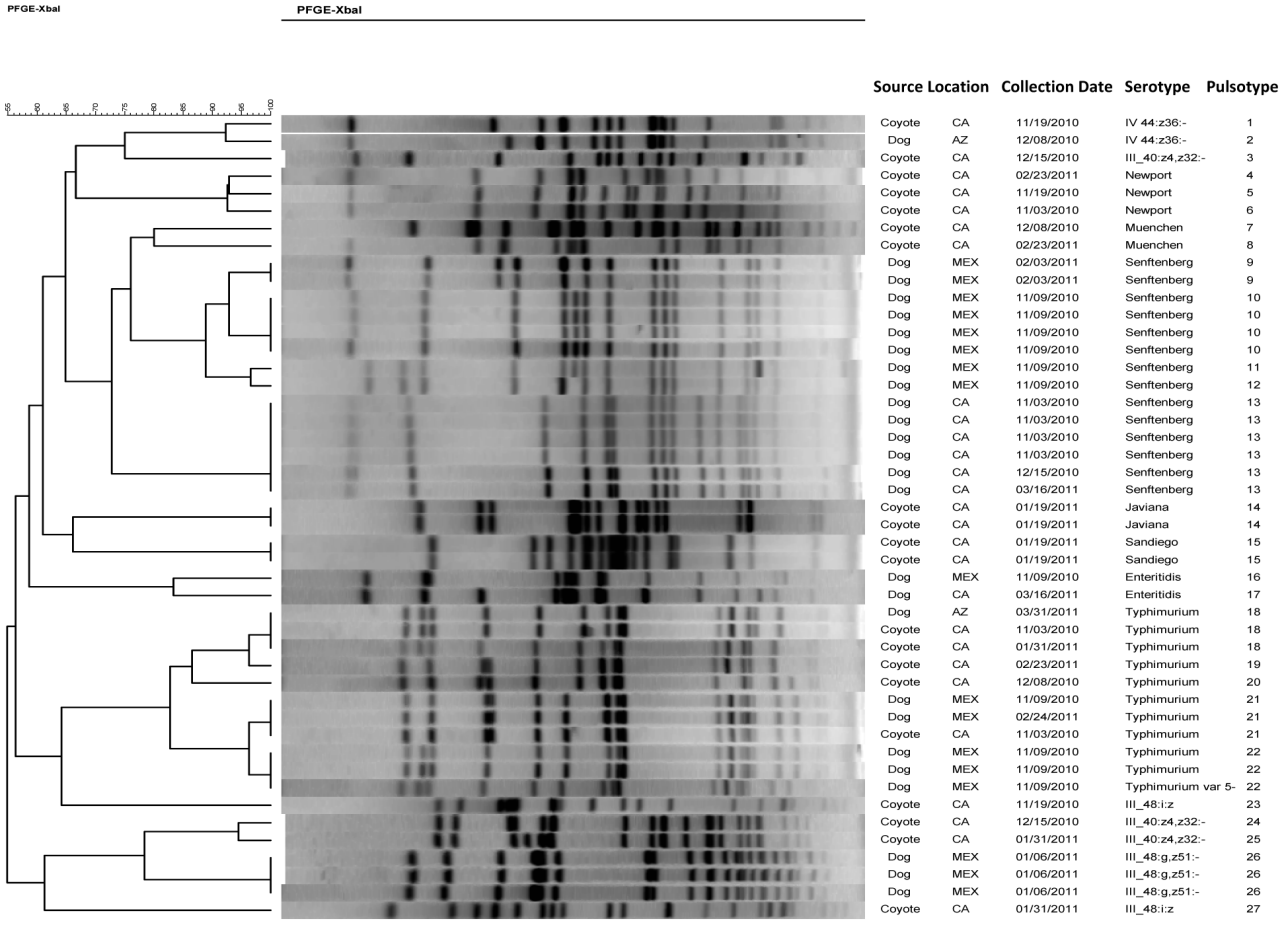
*Salmonella* Pulsotypes (*XbaI* restriction) isolated from dog and coyote fecal samples collected in the southwest desert produce growing areas of Arizona, California, and northern Mexico, November 3, 2010 through May 5, 2011.

As shown in Figure 3, PFGE analysis of *Salmonella* isolates revealed 27 distinct pulsotypes. *S*. Senftenberg isolates (n = 8) from dog feces collected on two dates at the shelter in Mexico belonged to four different but closely related pulsotypes (PT-9, 10, 11, 12). In contrast, *S*. Senftenberg isolates (n = 6) from California shelter dogs collected on three sampling dates belonged to a single pulsotype (PT-13). *S*. Typhimurium PT-18 and PT-21 were the only shared pulsotypes among samples from different locations and species including 2 California coyote and an Arizona dog isolate (PT-18), and two dogs from Mexico and a California coyote (PT-21).

Antibiotic resistance testing revealed that 58 (87.9%) of the 66 *Salmonella* isolates, evenly distributed between dogs and coyotes, were susceptible to the antibiotics tested (Table 5). Of the 8 *Salmonella* isolates with resistance to at least one antibiotic, 4 were resistant to 2 or more drugs. An *S*. Newport isolate from coyote feces collected in California showed the most antibiotic resistant phenotype in this study including resistance to ampicillin, amoxicillin/clavulanic acid, cefriaxone, chloramphenicol, and trimethoprim/sulfamethoxazole.

## Discussion

In this study, we show that stray dogs and free-roaming coyotes in the southwest desert leafy greens production region at the U.S.-Mexico border do not appear to be significant reservoirs of *E. coli* O157 and other STEC, but aEPEC and *Salmonella* were prevalent in fecal samples using the methods described herein.

### *E. coli* detection and characterization

Previous studies of STEC occurrence in domestic dogs in the U.S. have focused on detection of Shiga toxin genes among animals with and without gastroenteritis. In one survey, a higher prevalence of *stx1* (3% and 15%) and *stx2* (36% and 23%) was found in diarrheic and non-diarrheic greyhounds, respectively [34]. In another study, there was no occurrence of *stx1* or *stx2* in 52 healthy Midwestern research colony dogs [35]. For logistical reasons, we were not able to sample unhealthy dogs with diarrhea in isolation wards at the U.S. shelters, and health status information was not available at the shelter in Mexico; thus, we collected only fresh, normally formed fecal samples from dogs. Outside the U.S., a survey in Japan revealed an extremely low prevalence of *E. coli* O157:H7 in dogs and cats, with only 1 of 614 (0.2%) fecal samples testing positive [36]. If we over-estimated *E. coli* O157:H7 prevalence at 1% in our sample size calculations, it is possible we would have needed to collect more than 300 fecal samples to detect *E. coli* O157:H7 in the shelter dog population. In another Japanese survey, a positive association between the presence of dogs or cats on beef cattle farms and prevalence of O157 in cattle was found [37]. This association, however, could be attributed to the hygiene practices of the farm (e.g., farms that allow dogs to run loose may have poorer hygiene and biosecurity practices than farms that do not), rather than evidence of colonization. In Argentina, *stx1* and *stx2* were detected in 3.7% and 4.2% of dog samples, respectively, and STEC was culture confirmed in 4% of the samples [38]. While our results suggest that STEC is rare in southwest dog populations, there are caveats to consider when comparing our results with prevalence surveys in other locations. For example, in Argentina, dog rectal swabs were taken and screened for Shiga toxin genes, which may have lead to improved detection compared with our pen floor samples.

Data on STEC occurrence in coyotes is even sparser in the literature. *E. coli* O157:H7 prevalence surveys in the Midwest and Washington State found the pathogen in 0/100 and 0/7 coyote fecal samples, respectively [39], [40]. We tested a comparable number of coyote samples (n = 103), which may have been too few to detect *E. coli* O157:H7. In contrast, during a longitudinal study of various domestic and wild animals in the central California coast, *E. coli* O157:H7 was isolated from 2 of 145 (1.4%) colonic-fecal samples from hunted coyotes [19]. Interestingly, 2 (1.9%) of 103 coyote fecal samples in our study contained Shiga toxin-negative *E. coli* O157:H+ isolates positive for *eaeA.* Bentancor and colleagues (2010) have characterized non-Shiga toxin producing *E. coli* O157 strains from dogs in Argentina and speculated that strains with the *eae* gene may represent a potential human health threat [41].

The finding of aEPEC shedding in dog feces was not unexpected as others have isolated this pathotype in domestic dogs with and without diarrhea [42], [43], [44]. Typical EPEC is the leading cause of infantile diarrhea in developing countries, while atypical EPEC is considered an emerging zoonoses [32]. Indeed, it has been speculated that aEPEC is genetically related to STEC and isolates may share O:H serotypes [32], [33], [40]. In our study, we found several previously described aEPEC serotypes including O-:H2 (dog), O128:H2 (coyote), O145:H34 (dog), and O26:H11 (dog) (Table 4). Serotype O26:H11 is well-described in calves, while serotype O128:H2 is known to occur in dog and rabbit populations [32], [33], [45]. Interestingly, Trabulsi et al. (2002) suggest that O26:H11 and O128:H2 are not aEPEC, but rather a heterogeneous serotype of STEC and aEPEC [32].

We also endeavored to determine if the *E. coli* O145:H28 human clinical outbreak strain linked to Romaine lettuce grown in southern Arizona in spring 2010 was present in our fecal samples, but found that all strains in the *E. coli* O145 serogroup isolated during this study were phenotypically and genetically unrelated to the 2010 outbreak strain (Figure 2). The public health significance of Shiga toxin-negative *E.coli* strains belonging to serogroup O145, as well as the other Shiga toxin-negative isolates belonging to “top 6” STEC-associated serogroups found in our study, is still somewhat unclear. Ultimately, additional molecular studies are needed to better understand the human virulence potential of these strains. The most clinically significant strain we isolated may be Shiga toxin-negative *E. coli* O26:H11 *eaeA*+/*hlyA*+ cultured from dog feces at the Mexican shelter in February (Table 4). Loss and gain of *stx* genes by STEC O26 have been described, and aEPEC O26 may be ancestral to STEC O26 [46], [47]. We believe it is unlikely this strain or others lost Shiga toxin genes during passage through enrichment since *stx1*/*stx2* was not detected in TSB pre-enrichment broths by qPCR, provided the concentration of bacteria was within our level of detection. However, aEPEC strains could potentially re-acquire Shiga toxin genes and become STEC during passage through human or animal hosts [47].

We encountered several methodological challenges during this study related to culture and identification of STEC serogroups. For example, non-specific binding of STEC O-antigens (O103, O145, O157, O26) to IMS beads and method of capture may have caused isolation of multiple O-groups not originally targeted (Table 3). We also found discordant results between O-group specific latex agglutination and confirmatory tests, which has been described previously [48]. The utility of using a commercial latex agglutination screen to identify presumptive STEC from environmental samples such as feces needs further assessment and comparison with other methods including the serology gold standard. Nevertheless, we concluded that screening for Shiga toxin genes by qPCR would be a more efficient approach to detect STEC colonies in the future, rather than screening for STEC O-groups.

### *Salmonella* detection and characterization

Reported *Salmonella* prevalence in dogs varies greatly between studies, from 5% to over 70% [49], [50], [51], [52]. The predominance of two *Salmonella* serovars in the shelter dog population, but not in the sampled coyote population, is interesting. According to a USDA NVSL 2011 annual report, Typhimurium and Senftenberg, followed by Muenchen, Newport, and Javiana, were the most common serovars isolated nationally from dogs and cats during 2011 [53]. We speculate that the dominant serovars among shelter dog samples could indicate a common source of exposure to *Salmonella* in the environment. For example, contaminated dog food is increasingly recognized as a risk factor for *Salmonella* infections in dogs [54], [55], [56]. The findings could also be indicative of poor sanitation practices and overcrowding. Staffing at the facility in Mexico was noticeably limited and animals were typically kenneled in large groups of ten or more dogs, making the transmission of *Salmonella* between individuals more likely to occur. We controlled for this variable by limiting fecal collections to fresh feces from individual animals impounded within the last 24 hours. Often, however, it was noted that most of the individual animals sampled on a given day from the Mexican shelter had been collected from the same location and were likely living in groups together. Thus, dogs shedding genetically related strains may have shared common exposures prior to or during impound.

The fact that nearly one in three (32%) coyote fecal samples collected near leafy green fields were positive for *Salmonella* was surprising. In contrast, a survey from the central California coast found that the organism was recovered from only 3 (7.5%) of 40 coyote colonic fecal sample enrichment broths [11]. Variations in sampling and laboratory culture methods between the two studies could, in part, explain these differences. It is also possible that individual coyotes were re-sampled over the course of this study. However, given the wide geographic and temporal distribution of sampling locations in combination with the high diversity of serovars and PFGE subtypes (Figure 3), we do not believe that repeat sampling of individual coyotes contributed significantly to our overall prevalence. Even in the event of re-sampling, the apparent high prevalence of *Salmonella* in coyote fecal material found in or near the production area is of importance to growers given the potential to contaminate the plants directly, or indirectly via agriculture water sources and farm equipment, Additional studies using trapping or hunting techniques are needed to determine actual prevalence of foodborne pathogens in the southwest desert coyote population.

There was some seasonality observed in *Salmonella* recovery from both dog and coyote samples including a significantly higher prevalence in November compared with samples collected in late winter and spring months. More long-term studies, however, are indicated to reveal any true seasonality or temporal patterns of *Salmonella* occurrence. The relatively high prevalence of *Salmonella* shedding in dogs and coyotes may be due in part to the hunting/scavenging behaviors of canids in the region. In desert regions where prey and water resources are limited, both coyotes and stray dogs opportunistically forage on fresh and rotten nutritional sources, including garbage and other refuse, vegetable and fruit matter, and the meat of dead animals. Such scavenging behaviors may put dogs and coyotes at a higher risk of exposure to *Salmonella.* High prevalence of *Salmonella* has previously been found in other scavenging species of the southwest, such as turkey vultures (*Cathartes aura*) [57]. Additionally, these animals may be obtaining water from anthropogenic sources, such as irrigation ditches, sediment basins, and camp-sites in the absence of natural water sources. Water samples, from both static sources and flowing streams, rivers, and creeks, often yield high percentages of *Salmonella* positive samples [10], [58], [59].

We found more antibiotic resistance among aEPEC isolates compared with *Salmonella* isolates (Table 5). Four (22.2%) of 18 aEPEC and 4 (6.1%) of 66 *Salmonella* isolates were resistant to two or more antibiotic classes; two dog aEPEC isolates (O114:H8 and O167:H9), a dog *S*. Senftenberg, and a coyote *S*. Newport displayed resistance to 3 or more antibiotic classes. Of note, Newport and Senftenberg have been identified as emerging multi-drug-resistant serovars worldwide [60], [61]. In a wildlife study conducted during the same time period in the central California coast, a majority of *Salmonella enterica* subspecies Group IIIa and IIIb isolates from wild-caught amphibians and reptiles captured near produce fields were resistant to at least one antibiotic [12]. It appears that antibiotic resistance among *Salmonella* isolates is less prominent in canid populations tested in the southwest desert compared with the cold-blooded vertebrates surveyed in coastal California.

### Prevention and Control Recommendations

The produce industry currently addresses foodborne pathogen hazards from domestic and wild animal sources through adherence to best practices established by the Arizona and California Leafy Green Marketing Agreements [62], [63]. For example, fecal material in the production area is removed, and a minimal 5-foot radius no-harvest buffer zone (Figure 1D) is used to prevent contaminated plants from entering the food supply. Repeated intrusions into produce fields by dogs and coyotes can be managed by use of fencing, control of strays, and depredation (coyotes). In our study region, animal control officers on both sides of the border work closely with the produce growers to assist with stray animal problems in fields. In the Imperial-Yuma region, loose dogs can be particularly problematic in the fall-winter season—which is also the leafy greens growing season—when the area becomes a popular tourist (“snowbird”) destination for recreational vehicle enthusiasts who often travel with their dogs. In Mexico, stray dog control is more challenging because of limited resources, large numbers of un-owned dogs, and cultural barriers to dog population control.

It is worth noting that although intrusions by stray dogs may represent a food safety risk for fresh produce, trained working dogs if used properly can actually be an asset and should not be discouraged. For example, dogs have been used to help disperse and deter large flocks of nuisance birds, and scent detection dogs have been used experimentally to identify in-field fecal contamination of raw produce [64].

## Conclusions

In summary, results from this study will assist the produce industry by providing baseline information on the occurrence of zoonotic enteric pathogens found in fecal material from common domestic and wild canid populations in the desert southwest produce production region. The findings underscore the importance of good agriculture practices for leafy greens and other produce, especially those relating to animal intrusions and pre- and post-harvest environmental assessments. Follow-up surveys are warranted to determine pathogenic *E. coli* and *Salmonella* prevalence in other potential domestic and wildlife reservoirs in this region, and comparison with other possible environmental sources of microbial contamination (e.g., canals, irrigation water, and soil amendments).

## References

[1] Lynch MF, Tauxe RV, Hedberg CW (2009) The growing burden of foodborne outbreaks due to contaminated fresh produce: risks and opportunities. Epidemiol Infect 137: 307–315. 19200406 10.1017/S0950268808001969

[2] Berger CN, Sodha SV, Shaw RK, Griffin PM, Pink D, et al (2010) Fresh fruit and vegetables as vehicles for the transmission of human pathogens. Environ Microbiol 12: 2385–2397. 20636374 10.1111/j.1462-2920.2010.02297.x

[3] Lynch M, Painter J, Woodruff R, Braden C (2006) Surveillance for foodborne-disease outbreaks—United States, 1998–2002. MMWR Surveill Summ 55: 1–34. 17093388

[4] Sivapalasingam S, Friedman CR, Cohen L, Tauxe RV (2004) Fresh produce: a growing cause of outbreaks of foodborne illness in the United States, 1973 through 1997. J Food Prot 67: 2342–2353. 15508656 10.4315/0362-028x-67.10.2342

[5] Painter JA, Hoekstra RM, Ayers T, Tauxe RV, Braden CR, et al (2013) Attribution of foodborne illnesses, hospitalizations, and deaths to food commodities by using outbreak data, United States, 1998–2008. Emerg Infect Dis 19: 407–415. 23622497 10.3201/eid1903.111866 PMC3647642

[6] Mandrell RE (2011) Tracing pathogens in fruit and vegetable production chains. In: Brul S, Fratamico PM, McMeekin TA, editors. Tracing pathogens in the food chain.Cambridge: Woodhead Publishing Ltd. pp. 548–595.

[7] Jay-Russell MT (2013) What is the risk from wild animals in food-borne pathogen contamination of plants? CAB Reviews 8: 040.

[8] Boriss H, Brunke H. (2012) Produce Profile. Agricultural Issues Center, University of California. Available: http://www.agmrc.org/commodities__products/vegetables/lettuce-profile/. Accessed 18 October 2014.

[9] Jay MT, Cooley M, Carychao D, Wiscomb GW, Sweitzer RA, et al (2007) *Escherichia coli* O157:H7 in feral swine near spinach fields and cattle, central California coast. Emerg Infect Dis 13: 1908–1911. 18258044 10.3201/eid1312.070763 PMC2876768

[10] Cooley M, Carychao D, Crawford-Miksza L, Jay MT, Myers C, et al (2007) Incidence and tracking of *Escherichia coli* O157:H7 in a major produce production region in California. PloS ONE 2: e1159. 18174909 10.1371/journal.pone.0001159 PMC2174234

[11] Gorski L, Parker CT, Liang A, Cooley MB, Jay-Russell MT, et al (2011) Prevalence, distribution, and diversity of *Salmonella enterica* in a major produce region of California. Appl Environ Microbiol 77: 2734–2748. 21378057 10.1128/AEM.02321-10 PMC3126348

[12] Gorski L, Jay-Russell MT, Liang AS, Walker S, Bengson Y, et al (2013) Diversity of pulsed-field gel electrophoresis pulsotypes, serovars and antibiotic resistance among *Salmonella* isolates from wild amphibians and reptiles in the California central coast. Foodborne Pathog Dis 10: 540–548. 23577627 10.1089/fpd.2012.1372

[13] Kilonzo C, Li X, Vivas EJ, Jay-Russell MT, Fernandez KL, et al (2013) Fecal shedding of zoonotic food-borne pathogens by wild rodents in a major agricultural region of the central California coast. Appl Environ Microbiol 79: 6337–6344. 23934490 10.1128/AEM.01503-13 PMC3811224

[14] Strawn LK, Grohn YT, Warchocki S, Worobo RW, Bihn EA, et al (2013) Risk factors associated with *Salmonella* and *Listeria monocytogenes* contamination of produce fields. Appl Environ Microbiol 79: 7618–7627. 24077713 10.1128/AEM.02831-13 PMC3837806

[15] Taylor EV, Nguyen TA, Machesky KD, Koch E, Sotir MJ, et al (2013) Multistate outbreak of *Escherichia coli* O145 infections associated with Romaine lettuce consumption, 2010. J Food Prot 76: 939–944. 23726187 10.4315/0362-028X.JFP-12-503

[16] Cooper KK, Mandrell RE, Louie JW, Korlach J, Clark TA, et al (2014) Comparative genomics of enterohemorrhagic *Escherichia coli* O145:H28 demonstrates a common evolutionary lineage with *Escherichia coli* O157:H7. BMC Genomics 15: 17. 24410921 10.1186/1471-2164-15-17 PMC3893438

[17] Crawford W, Baloch M, Gerrity K (2010) Environmental assessment: non-O157 Shiga-toxin producing *E. coli* (STEC). U.S. Food and Drug Administration (FDA). Available: http://www.fda.gov/downloads/Food/RecallsOutbreaksEmergencies/UCM235923.pdf Accessed: 18 October 2014.

[18] Kanki M, Seto K, Sakata J, Harada T, Kumeda Y (2009) Simultaneous enrichment of shiga toxin-producing *Escherichia coli* O157 and O26 and *Salmonella* in food samples using universal preenrichment broth. J Food Prot 72: 2065–2070. 19833028 10.4315/0362-028x-72.10.2065

[19] Cooley MB, Jay-Russell M, Atwill ER, Carychao D, Nguyen K, et al (2013) Development of a robust method for isolation of Shiga toxin-positive *Escherichia coli* (STEC) from fecal, plant, soil and water samples from a leafy greens production region in California. PloS ONE 8: e65716. 23762414 10.1371/journal.pone.0065716 PMC3675059

[20] DebRoy C, Roberts E, Fratamico PM (2011) Detection of O antigens in *Escherichia coli* . Anim Health Res Rev 12: 169–185. 22152292 10.1017/S1466252311000193

[21] Machado J, Grimont F, Grimont PA (2000) Identification of *Escherichia coli* flagellar types by restriction of the amplified *fliC* gene. Res Microbiol 151: 535–546. 11037131 10.1016/s0923-2508(00)00223-0

[22] DebRoy C, Maddox C (2001) Assessing virulence of *Escherichia coli* isolates of veterinary significance. Anim Health Res Rev 1: 129–140. 11831435

[23] Barkocy-Gallagher GA, Berry ED, Rivera-Betancourt M, Arthur TM, Nou X, et al (2002) Development of methods for the recovery of Escherichia coli O157:H7 and *Salmonella* from beef carcass sponge samples and bovine fecal and hide samples. J Food Prot 65: 1527–1534. 12380735 10.4315/0362-028x-65.10.1527

[24] Grimot PAD, Weill FX (2007) Antigenic formulae of the *Salmonella* serovars, 9^th^ revision. World Health Organization Collaborating Centre for Reference and Research on *Salmonella*. Paris: Pasteur Institute.

[25] Ribot EM, Fair MA, Gautom R, Cameron DN, Hunter SB, et al (2006) Standardization of pulsed-field gel electrophoresis protocols for the subtyping of *Escherichia coli* O157:H7, *Salmonella*, and *Shigella* for PulseNet. Foodborne Path Dis 3: 59–67. 10.1089/fpd.2006.3.59 16602980

[26] Tenover FC, Arbeit RD, Goering RV, Mickelsen PA, Murray BE, et al (1995) Interpreting chromosomal DNA restriction patterns produced by pulsed-field gel electrophoresis: criteria for bacterial strain typing. J Clin Microbiol 33: 2233–2239. 7494007 10.1128/jcm.33.9.2233-2239.1995 PMC228385

[27] Barrett TJ, Gerner-Smidt P, Swaminathan B (2006) Interpretation of pulsed-field gel electrophoresis patterns in foodborne disease investigations and surveillance. Foodborne Pathog Dis 3: 20–31. 16602976 10.1089/fpd.2006.3.20

[28] Anonymous (2008) Performance standards for antimicrobial disk and dilution susceptibility tests for bacteria isolated from animals; Approved Standard—Third Edition. Wayne, PA: Clinical and Laboratory Standards Institute CLSI document M31–A3.

[29] Anonymous (2008) Performance standards for antimicrobial susceptibility testing; Twentieth Informational Supplement. Wayne, PA: Clinical and Laboratory Standards Institute CLSI document M100–S20.

[30] Anonymous (2012) National Antimicrobial Resistance Monitoring System– Enteric Bacteria (NARMS): 2010 Executive Report. National Antimicrobial Resistance Monitoring System– Enteric Bacteria (NARMS): 2010 Executive Report. U.S. Food and Drug Administration. Available: http://www.fda.gov/AnimalVeterinary/SafetyHealth/AntimicrobialResistance/NationalAntimicrobialResistanceMonitoringSystem/ucm312356.htm. Accessed 18 October 2014.

[31] Sjolund-Karlsson M, Joyce K, Blickenstaff K, Ball T, Haro J, et al (2011) Antimicrobial susceptibility to azithromycin among *Salmonella enterica* isolates from the United States. Antimicrob Agents Chemother 55: 3985–3989. 21690279 10.1128/AAC.00590-11 PMC3165283

[32] Trabulsi LR, Keller R, Tardelli Gomes TA (2002) Typical and atypical enteropathogenic *Escherichia coli* . Emerg Infect Dis 8: 508–513. 11996687 10.3201/eid0805.010385 PMC2732489

[33] Bugarel M, Martin A, Fach P, Beutin L (2011) Virulence gene profiling of enterohemorrhagic (EHEC) and enteropathogenic (EPEC) *Escherichia coli* strains: a basis for molecular risk assessment of typical and atypical EPEC strains. BMC Microbiol 11: 142. 21689465 10.1186/1471-2180-11-142 PMC3133550

[34] Staats JJ, Chengappa MM, DeBey MC, Fickbohm B, Oberst RD (2003) Detection of *Escherichia coli* Shiga toxin (*stx*) and enterotoxin (*estA* and *elt*) genes in fecal samples from non-diarrheic and diarrheic greyhounds. Vet Microbiol 94: 303–312. 12829384 10.1016/s0378-1135(03)00134-2

[35] Holland RE, Walker RD, Sriranganathan N, Wilson RA, Ruhl DC (1999) Characterization of *Escherichia coli* isolated from healthy dogs. Vet Microbiol 70: 261–268. 10596809 10.1016/s0378-1135(99)00136-4

[36] Kataoka Y, Irie Y, Sawada T, Nakazawa M (2010) A 3-year epidemiological surveillance of *Escherichia coli* O157:H7 in dogs and cats in Japan. J Vet Med Sci 72: 791–794. 20124764 10.1292/jvms.09-0439

[37] Sasaki Y, Tsujiyama Y, Kusukawa M, Murakami M, Katayama S, et al (2011) Prevalence and characterization of Shiga toxin-producing *Escherichia coli* O157 and O26 in beef farms. Vet Microbiol 150: 140–145. 21292409 10.1016/j.vetmic.2010.12.024

[38] Bentancor A, Rumi MV, Gentilini MV, Sardoy C, Irino K, et al (2007) Shiga toxin-producing and attaching and effacing *Escherichia coli* in cats and dogs in a high hemolytic uremic syndrome incidence region in Argentina. FEMS Microbiol Lett 267: 251–256. 17328115 10.1111/j.1574-6968.2006.00569.x

[39] Renter DG, Sargeant JM, Oberst RD, Samadpour M (2003) Diversity, frequency, and persistence of *Escherichia coli* O157 strains from range cattle environments. Appl Environ Microbiol 69: 542–547. 12514039 10.1128/AEM.69.1.542-547.2003 PMC152399

[40] Rice DH, Hancock DD, Besser TE (2003) Faecal culture of wild animals for *Escherichia coli* O157:H7. Vet Rec 152: 82–83. 12570312 10.1136/vr.152.3.82

[41] Bentancor A, Vilte DA, Rumi MV, Carbonari CC, Chinen I, et al (2010) Characterization of non-Shiga-toxin-producing *Escherichia coli* O157 strains isolated from dogs. Rev Argent Microbiol 42: 46–48. 20461294 10.1590/S0325-75412010000100010

[42] Goffaux F, China B, Janssen L, Mainil J (2000) Genotypic characterization of enteropathogenic *Escherichia coli* (EPEC) isolated in Belgium from dogs and cats. Res Microbiol 151: 865–871. 11191812 10.1016/s0923-2508(00)01153-0

[43] Marks SL, Rankin SC, Byrne BA, Weese JS (2011) Enteropathogenic bacteria in dogs and cats: diagnosis, epidemiology, treatment, and control. J Vet Intern Med 25: 1195–1208. 22092607 10.1111/j.1939-1676.2011.00821.x

[44] Puno-Sarmiento J, Medeiros L, Chiconi C, Martins F, Pelayo J, et al (2013) Detection of diarrheagenic *Escherichia coli* strains isolated from dogs and cats in Brazil. Vet Microbiol 166: 676–680. 23932311 10.1016/j.vetmic.2013.07.007

[45] Moura RA, Sircili MP, Leomil L, Matte MH, Trabulsi LR, et al (2009) Clonal relationship among atypical enteropathogenic *Escherichia coli* strains isolated from different animal species and humans. Appl Environ Microbiol 75: 7399–7408. 19801470 10.1128/AEM.00636-09 PMC2786407

[46] Bielaszewska M, Middendorf B, Kock R, Friedrich AW, Fruth A, et al (2008) Shiga toxin-negative attaching and effacing *Escherichia coli*: distinct clinical associations with bacterial phylogeny and virulence traits and inferred in-host pathogen evolution. Clin Infect Dis 47: 208–217. 18564929 10.1086/589245

[47] Bielaszewska M, Prager R, Kock R, Mellmann A, Zhang W, et al (2007) Shiga toxin gene loss and transfer in vitro and in vivo during enterohemorrhagic *Escherichia coli* O26 infection in humans. Appl Environ Microbiol 73: 3144–3150. 17400784 10.1128/AEM.02937-06 PMC1907125

[48] Kimura R, Mandrell RE, Galland JC, Hyatt D, Riley LW (2000) Restriction-site specific PCR as a rapid test to detect enterohemorrhagic *Escherichia coli* O157:H7 strains in environmental samples. Appl Environ Microbiol 66: 2513–2519. 10831431 10.1128/aem.66.6.2513-2519.2000 PMC110571

[49] Joffe DJ, Schlesinger DP (2002) Preliminary assessment of the risk of *Salmonella* infection in dogs fed raw chicken diets. Can Vet J 43: 441–442. 12058569 PMC339295

[50] McKenzie E, Riehl J, Banse H, Kass PH, Nelson S Jr, et al (2010) Prevalence of diarrhea and enteropathogens in racing sled dogs. J Vet Intern Med 24: 97–103. 19925573 10.1111/j.1939-1676.2009.0418.x

[51] Lenz J, Joffe D, Kauffman M, Zhang Y, LeJeune J (2009) Perceptions, practices, and consequences associated with foodborne pathogens and the feeding of raw meat to dogs. Can Vet J 50: 637–643. 19721784 PMC2684052

[52] Leonard EK, Pearl DL, Finley RL, Janecko N, Peregrine AS, et al (2011) Evaluation of pet-related management factors and the risk of *Salmonella* spp. carriage in pet dogs from volunteer households in Ontario (2005–2006). Zoonoses Public Health 58: 140–149. 20163574 10.1111/j.1863-2378.2009.01320.x

[53] Lantz K (2012) *Salmonella* serotypes isolated from animals in the United States: January 1 – December 31, 2011. Report of the Committee on *Salmonella*, United States Animal Health Association. Available: http://www.usaha.org/Portals/6/Reports/2012/report-sal-2012.pdf. Accessed 18 October 2014.

[54] Behravesh CB, Ferraro A, Deasy M 3rd, Dato V, Moll M, et al (2010) Human *Salmonella* infections linked to contaminated dry dog and cat food, 2006–2008. Pediatrics 126: 477–483. 20696725 10.1542/peds.2009-3273

[55] Anonymous (2008) Multistate outbreak of human *Salmonella* infections caused by contaminated dry dog food—United States, 2006-2007. MMWR Morbid MortalWeekly Rep 57: 521–524. 18480745

[56] Selmi M, Stefanelli S, Bilei S, Tolli R, Bertolotti L, et al (2011) Contaminated commercial dehydrated food as source of multiple *Salmonella* serotypes outbreak in a municipal kennel in Tuscany Vet Ital. 47: 183–190. 21706471

[57] Winsor DK, Bloebaum AP, Mathewson JJ (1981) Gram-negative, aerobic, enteric pathogens among intestinal microflora of wild turkey vultures (*Cathartes aura*) in west central Texas. Appl Environ Micrbiol 42: 1123–1124. 10.1128/aem.42.6.1123-1124.1981 PMC244163 7032423

[58] Economou V, Gousia P, Kansouzidou A, Sakkas H, Karanis P, et al (2013) Prevalence, antimicrobial resistance and relation to indicator and pathogenic microorganisms of *Salmonella* enterica isolated from surface waters within an agricultural landscape. Int J Hyg Environ Health 216: 435–444. 22901425 10.1016/j.ijheh.2012.07.004

[59] Wilkes G, Edge TA, Gannon VP, Jokinen C, Lyautey E, et al (2011) Associations among pathogenic bacteria, parasites, and environmental and land use factors in multiple mixed-use watersheds. Water Res 45: 5807–5825. 21889781 10.1016/j.watres.2011.06.021

[60] Whichard JM, Gay K, Stevenson JE, Joyce KJ, Cooper KL, et al (2007) Human *Salmonella* and concurrent decreased susceptibility to quinolones and extended-spectrum cephalosporins. Emerg Infect Dis 13: 1681–1688. 18217551 10.3201/eid1311.061438 PMC3375806

[61] Anonymous (2005) Drug-resistant *Salmonella* World Health Organization. Available: http://www.who.int/mediacentre/factsheets/fs139/en/ Accessed 18 October 2014.

[62] Anonymous (2013) Arizona Leafy Green Marketing Agreement. Available: http://www.arizonaleafygreens.org/. Accessed 18 October 2014.

[63] Anonymous (2013) California Leafy Green Marketing Agreement. Available: http://www.caleafygreens.ca.gov/. Accessed 18 October 2014.

[64] Partyka ML, Bond RF, Farrar J, Falco A, Cassens B, et al (2014) Quantifying the sensitivity of scent detection dogs to identify fecal contamination on raw produce. J Food Prot 77: 6–14. 24405993 10.4315/0362-028X.JFP-13-249

